# Oncogenic microRNA signature for early diagnosis of cervical intraepithelial neoplasia and cancer

**DOI:** 10.1002/1878-0261.12383

**Published:** 2018-09-27

**Authors:** Stephanie S. Liu, Karen K. L. Chan, Daniel K. H. Chu, Tina N. Wei, Lesley S. K. Lau, Siew F. Ngu, Mandy M. Y. Chu, Ka Yu Tse, Philip P. C. Ip, Enders K. O. Ng, Annie N. Y. Cheung, Hextan Y. S. Ngan

**Affiliations:** ^1^ Department of Obstetrics & Gynaecology LKS Faculty of Medicine The University of Hong Kong SAR China; ^2^ Department of Pathology LKS Faculty of Medicine Queen Mary Hospital The University of Hong Kong SAR China; ^3^ Pangenia Lifesciences Limited Billion Centre Hong Kong SAR China

**Keywords:** cervical cancer, cervical intraepithelial neoplasia, diagnostic biomarker, human papillomavirus, microRNA, sensitivity and specificity

## Abstract

Cervical cancer is one of the leading causes of cancer death in women globally, despite the widespread use of cytology/human papillomavirus (HPV) screening. In the present study, we aimed to identify the potential role of microRNA (miRNA) as a diagnostic biomarker in the detection of cervical pre‐malignant lesions and cancer. In total, we recruited 582 patients with cervical diseases and 145 control individuals. The expression levels of six miRNAs (miR‐20a, miR‐92a, miR‐141, miR‐183*, miR‐210 and miR‐944) were found to be significantly up‐regulated in cervical cancer and pre‐malignant lesions compared to normal cervical samples, indicating that they are oncogenic miRNAs. Receiver operating characteristic curve analysis showed that these six miRNAs can be used to distinguish patients with cervical pre‐malignant lesions or cancer from normal individuals and they also had a good predictive performance, particularly in cervical lesions. Combined use of these six miRNAs further enhanced the diagnostic accuracy over any single miRNA marker, with an area under the curve of 0.998, 0.996 and 0.959, a diagnostic sensitivity of 97.9%, 97.2% and 91.4%, and a specificity of 98.6%, 96.6% and 87.6% for low‐grade lesions, high‐grade lesions and cancer, respectively. This six oncogenic miRNA signature may be suitable for use as diagnostic marker for cervical pre‐malignant lesions and cancer in the near future.

**Abbreviations**
AUC: area under the curve
hrHPV: high‐risk human papillomavirus
LG and HG‐CIN: low‐grade and high‐grade cervical intraepithelial neoplasia
miRNA: microRNA
ROC: Receiver operating characteristic

## 1 Introduction

Cervical cancer is the third most common cancer in women worldwide and is ranked second in Asia (Forouzanfar *et al*., 2011). It is associated with high mortality and morbidity. The high‐risk human papillomavirus (HPV) infection is the most important etiological agent for cervical cancer (Schiffman *et al*., 2007). The development of cervical cancer is a multistep process occurring over a period of years, from the transformation of the normal cervical epithelium to pre‐malignant lesions (known as cervical intraepithelial neoplasia; CIN) and subsequently to invasive cancer. The clinical outcome of cancer patients is mainly dependent on the clinical stage at which therapy is initiated, and patients with cancer at an advanced stage have a poor overall survival. Cervical pre‐malignant lesions are usually curable; therefore, the detection of a cervical lesion at its early stage will facilitate a treatment strategy to improve the clinical outcome, subsequently reducing the incidence of cervical cancer and mortality rates. Recent cervical cancer screening relies on cytology/HPV testing. Although the cytology‐based cervical cancer screening program has reduced the incidence of cervical cancer in many developed countries, the cytology test has limited sensitivity and a costly infrastructure (Peto *et al*., 2004). The etiological association of high‐risk HPV (hrHPV) infection with the development of cervical cancer has led to the introduction of hrHPV DNA/RNA testing for cervical cancer screening. Accumulated evidence shows that HPV testing offers a higher sensitivity but lower specificity than the cytology test in the identification of high‐grade CIN (Mayrand *et al*., 2007). Therefore, an effective tool with both high sensitivity and specificity for cervical cancer screening is currently lacking. The identification of novel molecular markers may provide alternative screening tool for the early detection and diagnosis of cervical lesions and/or cancer.

MicroRNAs (miRNAs) are small noncoding regulatory RNAs of 21–25 nucleotides in length. They are integral to the gene regulatory network by regulating cellular gene expression at the post‐transcriptional level through base pairing with targeted mRNAs to induce mRNA degradation or translational suppression. miRNAs are differentially expressed and target approximately 60% of mRNA targets to modulate a wide spectrum of biological processes (Lin and Gregory, 2015). Therefore, miRNAs have been implicated in the etiology and pathogenesis of human cancers, and aberrant miRNA expression is common in most of human cancers. Previous studies have demonstrated that specific miRNA expression signatures were associated with clinical diagnosis, prognosis or treatment responses in a number of human cancers (Bertoli *et al*., 2015).

The present study aimed to explore the clinical value of aberrant miRNA expression as potential biomarkers in the diagnosis and disease progression of cervical pre‐malignant lesions and cancer.

## 2 Materials and methods

### 2.1 Study design, patients and samples

**Figure 1:**
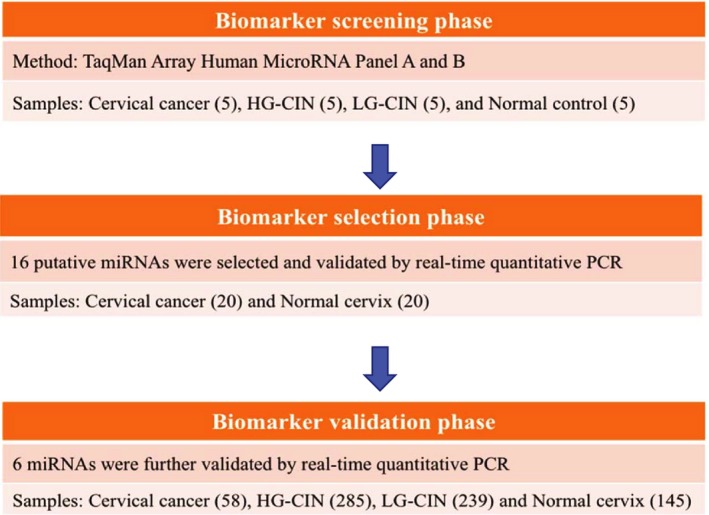
Schematic diagram of the study workflow.

The study was approved by the institutional review board of the University of Hong Kong/Hospital Authority Hong Kong West Cluster (IRB HKU/HA HKWC, No. UW05‐143T/806 and UW11‐251) and the experiments were undertaken with the informed written consents of all participants. The study methodologies conformed with the standards set by the Declaration of Helsinki. A multiphase case–control study was designed to identify diagnostic miRNA markers for cervical intraepithelial neoplasia and cancer (Fig. 1). Study subjects were recruited at Department of Obstetrics and Gynaecology, University of Hong Kong, Queen Mary Hospital. Two hundred and three cervical tissue samples were retrieved between 2006 and 2013, including 58 cervical cancers and 145 normal cervical tissues. Cervical tissues were obtained immediately after the surgical procedure and stored in liquid nitrogen. The histology of cancer samples was confirmed, and tumor contents were never less than 70%. All normal cervical tissues were obtained from women undergoing hysterectomy or colposcopy for benign diseases. In addition, 524 cervical CIN samples were archived paraffin‐embedded tissues and retrospectively retrieved from Department of Pathology, University of Hong Kong, Queen Mary Hospital. The hemotoxylin and eosin sections of CIN samples were reviewed by a pathologist to confirm the diagnosis.

Relevant clinical data regarding patient demographics, survival and tumor pathology were retrieved from their medical records for correlation analyses.

### 2.2 miRNA expression profiling

In the initial screening phase, miRNA expression was profiled using TagMan Array Human MicroRNA Panel A and B (Applied Biosystems, Foster City, CA, USA) on a set of cervical samples, including normal cervical tissue, as well as low‐grade (LG) and high‐grade (HG)‐CIN and cervical cancer, with five samples in each group. The TaqMan‐based quantitative PCR (qPCR) array was performed using TaqMan Universal PCR Master Mix in an ABI PRISM 7900 HT system (Applied Biosystems) in accordance with the manufacturer's instructions.

### 2.3 Total RNA extraction and miRNA detection

The total RNA containing miRNA from frozen cervical cancer or normal cervical tissues was extracted and purified using miRNeasy Mini Kit (Qiagen, Hilden, Germany). CIN sections were macro‐dissected from a paraffin‐embedded block and total RNA was extracted using the miRNeasy FFPE Kit (Qiagen). cDNA synthesis was carried out from 1 μg of total RNA in a reaction volume of 10 μl with miScript II RT Kit (Qiagen) in accordance with the manufacturer's instructions.

Putative miRNA markers selected from the miRNA profiling study were quantitated and validated in cervical cancers, CIN and normal cervical tissues using real‐time qPCR with miScript SYBR Green PCR Kit (Qiagen) on an ABI ViiA7 system (Applied Biosystems). Amplification was performed with the miScript Universal primer provided by manufacture and the miRNA‐specific forward primer designed based on the miRNA sequences obtained from the miRBase database (http://www.mirbase.org). All of the reactions were run in duplicate and the *C*_t_ values were determined using the fixed threshold settings. RNU6B (U6 small nuclear RNA) was used as an internal reference for normalization of the expression level of candidate miRNAs. To confirm RNU6B as a reliable internal normalizer, the expression level of RNU6B had been evaluated previously in a subset of tissue samples of 20 normal cervical tissues, 30 CINs and 20 cervical cancers. No significant difference (*C*_t_ values) was found between three groups of samples (*P* = 1.0, *P* = 0.26 and *P* = 0.08 for normal versus CIN, normal versus cancer and CIN versus cancer) (Fig. S1A), suggesting that RNU6B uniformly expressed in cervical tissue samples regardless of different disease status. Similar findings of using RNU6B as internal reference in miRNA expression studies in cervical lesions and cancer were also reported (Honegger *et al*., 2015; Park *et al*., 2017; Tian *et al*., 2014). After the relative quantification of miRNA expression was calculated, the fold change of the miRNA was analyzed using: $2^{-\Delta \Delta C_{t}}$. Δ*C*_t_ was calculated by subtracting the average *C*_t_ values of U6 from the *C*_t_ value of miRNA of interested. ΔΔ*C*_t_ was then calculated by subtracting Δ*C*_t_ of the control from Δ*C*_t_ of cancer or CIN.

It is suggested that miR‐25‐3p and miR‐93‐5p should also be used as internal normalizers because they were previously identified as suitable endogenous controls in human cancer cell lines, as reported by Das *et al*. (2016). We then detected the expression levels of these two miRNAs in part of our samples, including 20 normal cervical tissues, 30 CINs and 20 cervical cancers. As shown in Fig. S1B,C, the expression of both miRNAs increased gradually from CIN to cancer. There were significant differences in the mean *C*_t_ value of miR‐93‐5p between three groups of samples (*P* = 0.04, *P* < 0.0001 and *P* = 0.0001 for normal versus CIN, normal versus cancer and CIN versus cancer). Similarly, the mean *C*_t_ value of miR‐25‐3p was also significantly different between normal, CIN and cancer, although not between normal and CIN (*P* < 0.0001, *P* = 0.0001 and *P* = 0.23 for normal versus CIN, normal versus cancer and CIN versus cancer). Our results indicated that miR‐25‐3p and miR‐93‐5p expressed inconsistently in cervical tissue samples with different disease status, suggesting that they might not be suitable internal normalizers for investigating miRNA in cervical tissue samples.

### 2.4 DNA extraction and HPV genotyping

Genomic DNA was extracted from frozen cervical cancer and normal tissues using a conventional phenol/chloroform method and from paraffin‐embedded CIN tissues using QIAamp DNA FFPE Tissue Kit (Qiagen). hrHPV was detected and genotyped using the Hybribio HPV GenoArray test (Hybribio Ltd, Sheung Wan, Hong Kong) or the Hybrid Capture II assay (Qiagen) for DNA extracted from Frozen tissues, as well as the INNO‐LiPA HPV Genotyping Extra Kit (Fujirebio, Gent, Belgium) for DNA extracted from paraffin samples.

### 2.5 Target gene prediction and enrichment analysis

The potential target genes of candidate miRNAs were predicted through three different online algorithms: miRDB (http://www.mirdb.org/miRDB) (Wong and Wang, 2015); TargetScan v7.1 (http://www.targetscan.org) (Agarwal *et al*., 2015); and DIANA microT‐CDS Web Server v5.0 (http://www.microrna.gr/micrT-CDS) (Paraskevopoulou *et al*., 2013). To further strengthen the reliability of the bioinformatics analysis, the overlapping target genes from three online tools of each miRNA were identified using a Venn diagram (RStudio, 2018). The pathway enrichment analysis of overlapping target genes was conducted through The Database for Annotation, Visualization and Integrated Discovery (DAVID v6.7) bioinformatics online tool (http://david.nicfcrf.gov). *P* < 0.05 was set as the cut‐off criteria.

### 2.6 Statistical analysis

Statistical analysis was performed using spss, version 22 (IBM Corp., Armonk, NY, USA) and Prism, version 6 (GraphPad Software Inc., San Diego, CA, USA). The significance of miRNA expression level was determined using the Mann–Whiney *U*‐test (for nonparametric data comparison between two groups) and the Kruskal–Wallis test with Dunn's multiple comparison post‐hoc test (for nonparametric data comparison between three or more groups). The median miRNA intensity value of each cohort was used as the cut‐off point to categorize patients into groups with high or low expression for clinical correlation analysis. Pearson's chi‐squared test was used to examine the associations between miRNA expression level and clinicopathological parameters of patients with cervical cancer or CIN. Kaplan–Meier analysis was applied to estimate patient overall and disease progressive free survival, and the log‐rank test was used to assess survival difference between two groups. Binary logistic regression analysis was used to generate a comprehensive set of multi‐miRNA markers (combined six miRNA markers) for evaluation. Receiver operating characteristic (ROC) curves and the area under the curve (AUC) for each (or combined) miRNA marker were constructed and calculated with the 95% confidence interval (CI) to assess the performance of miRNA detection with respect to identifying patients with CIN or cancer. The optimal cut‐off point, diagnostic sensitivity and specificity, and positive and negative predictive value from ROC curves were determined by a commonly used method with 95% CI. *P *<* *0.05 (two‐sided) was considered statistically significant.

## 3 Results and Discussion

### 3.1 Clinicopathological characteristics of patients

In total, 582 patients and 145 normal controls were recruited in the present study and their clinical and pathological characteristics are summarized in Table 1. All cervical cancer samples were in early disease stage. hrHPV infection was detected in most of the cancer samples (55 of 58). Thirty‐five out of 145 normal cervical tissue samples were found to be hrHPV positive. Paraffin‐embedded CIN samples were obtained from 239 and 285 patients with LG‐ or HG‐CIN, respectively. hrHPVs were detected in 154 and 235 LG‐ and HG‐CIN samples. There were insufficient samples for HPV detection in 27 LG‐CIN and eight HG‐CIN samples.

**Table 1:** Clinical characteristics of patients and normal controls

Characteristic	Cervical cancer (*n* = 58)	HG‐CIN (*n* = 285)	LG‐CIN (*n* = 239)	Normal control (*n* = 145)
Age (years)				
Mean (range)	51 (30–81)	42 (19–87)	39 (19–79)	50 (30–88)
Disease stage				
1B	46			
2A	12			
Tumor histology				
SCC	42			
AD	10			
AS	5			
Others	1			
Tumor grade				
Grade 1	3			
Grade 2	28			
Grade 3	25			
Unknown	2			
HPV status				
hrHPV positive	55	235	153	35
lrHPV positive		10	9	
HPV negative	3	32	50	110
Unknown		8	27	

### 3.2 Discovery of cervical cancer‐related miRNA markers

**Figure 2:**
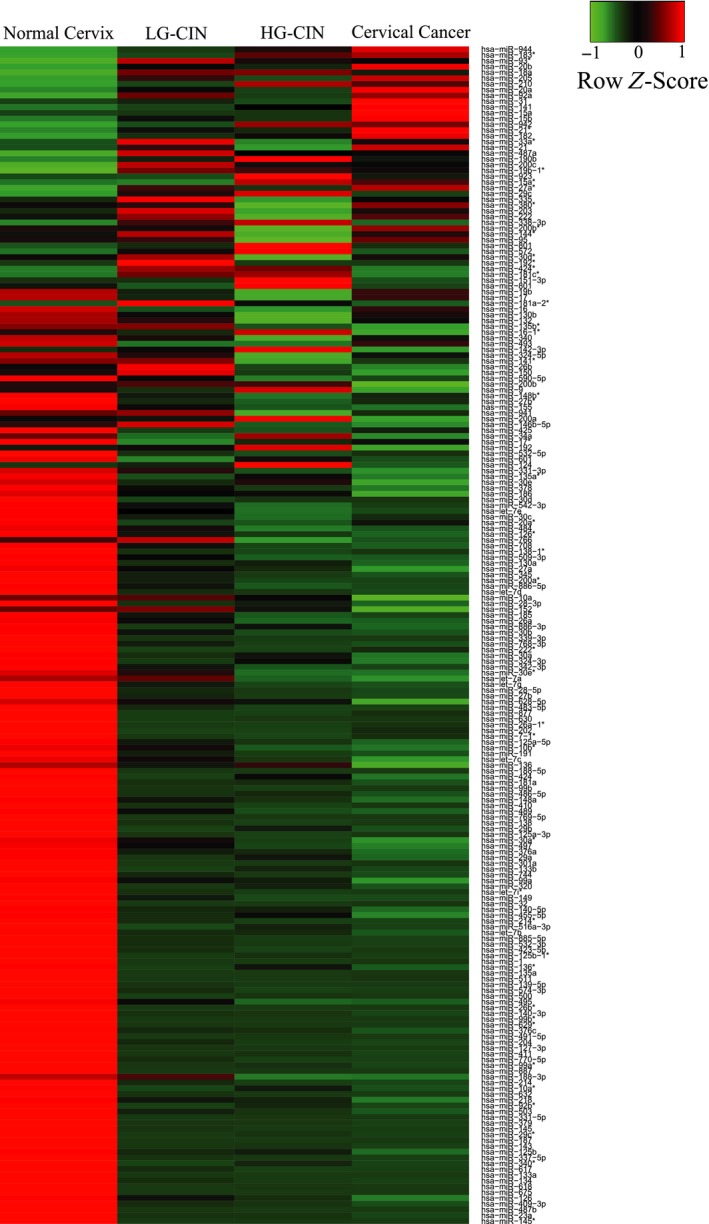
A heat map diagram of the miRNA expression profile of cervical lesions and cancer. The columns represent the study groups and rows represent the differential expression of miRNAs. Red indicates high expression, whereas green indicates low expression.

miRNA microarray profiling showed a panel of differentially expressed miRNAs between normal controls, LG and HG‐CINs and cancers (Fig. 2). Because we were interested in those miRNAs with up‐regulated expression in cancer, 16 miRNAs (on the top rows of the heat mat diagram) were selected and validated in a small set of cervical cancers and normal cervical tissues (20 samples in each group) using real‐time PCR. Subsequently, the expression of six out of 16 validated markers (miR‐20a, miR‐92a, miR‐141, miR‐183*, miR‐210 and miR‐944) was confirmed and selected for further evaluation in the present study (Fig. 1).

**Figure 3:**
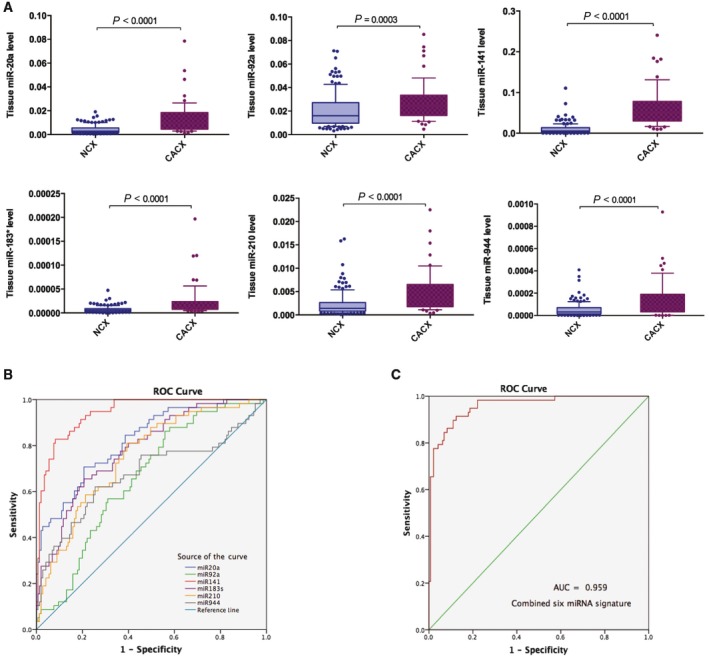
Differential expression of six miRNAs in cervical cancer and normal cervical tissues, and ROC analyses of single/combined six miRNA detection. (A) Box plot of miRNA expression in tissues (upper: miR‐20a, miR‐92a and miR‐141; lower: miR‐183*, miR‐210 and miR‐944) are normalized to RNU6B. The lines inside the boxes indicate the medians. The boxes mark the interval between the 25th and 75th percentiles. The whiskers and filled circles indicate the interval between and outside the 10th and 90th percentiles. A statistically significant difference is determined by the Mann–Whitney test. NCX, normal cervical tissue; CACX, cervical cancer. (B) ROC of six single miRNA detections. (C) ROC of combined six miRNA detection.

As shown in Fig. 3A, differential expression of six miRNAs was observed between cervical cancers and normal controls. The relative expression of six miRNAs was significantly up‐regulated in cancer samples (*P* < 0.0001, except for miR‐92a, *P* = 0.0003) compared to those in normal controls, indicating that they are oncogenic miRNAs associated with cervical carcinogenesis. We intended to select all early stage cervical cancer samples in the present study becasuse we aimed to explore those miRNAs with dysregulated expression in early disease stage.

To evaluate the diagnostic value of these miRNA markers in cervical cancer, ROC analysis was performed (Fig. 3B,C). The details of the AUC, sensitivity and specificity of each miRNA marker are listed in Table 2. The AUC of six miRNAs ranged from 0.659 to 0.942, indicating that all six miRNA markers were able to distinguish cancer patients from normal individuals (Fig. 3B). The sensitivities and specificities of miRNA markers to detect patient with cervical cancer ranged from 56.9% to 82.8% and 60.7% to 91.7%, respectively. Among the six miRNAs studied, miRNA‐141 is the best marker for differentiating the cancer patients (AUC = 0.942) with the highest sensitivity (82.8%) and specificity (91.7%). In an attempt to improve the miRNA detection efficacy, we constructed a binary logistic regression model to evaluate the performance of the combined use of six miRNA markers. The results showed that the diagnostic performance of combined use of miRNAs was improved significantly over any single miRNA marker and achieved an excellent AUC of 0.959, a sensitivity of 91.4%, a specificity of 87.6%, a positive predicative value (PPV) of 74.6% and a negative predicative value (NPV) of 96.2% (Fig. 3C and Table 2).

**Table 2:** The performance efficiency of miRNA detections as diagnostic markers in cervical cancer

Cervical cancer					
Marker	AUC (95% CI)	Sensitivity (95% CI)	Specificity (95% CI)	PPV % (95% CI)	NPV % (95% CI)
miR‐20a	0.822 (0.760–0.885)	70.7 (57.3–81.9)	79.3 (71.8–85.6)		
miR‐92a	0.659 (0.582–0.737)	56.9 (43.2–69.8)	69.0 (60.8–76.4)		
miR‐141	0.942 (0.912–0.973)	82.8 (70.6–91.4)	91.7 (86.0–95.7)		
miR‐183*	0.785 (0.717–0.852)	65.5 (51.9–77.5)	79.3 (71.8–85.6)		
miR‐210	0.751 (0.679–0.822)	81.0 (68.6–90.1)	60.7 (52.2–68.7)		
miR‐944	0.677 (0.586–0.769)	62.0 (48.4–74.5)	74.5 (66.6–81.4)		
Combined six miRNA signature	0.959 (0.932–0.987)	91.4 (81.0–97.1)	87.6 (81.1–92.5)	74.6 (63.1–86.2)	96.2 (92.5–99.9)

### 3.3 Evaluation of six miRNA signature in cervical intraepithelial neoplasia

**Figure 4:**
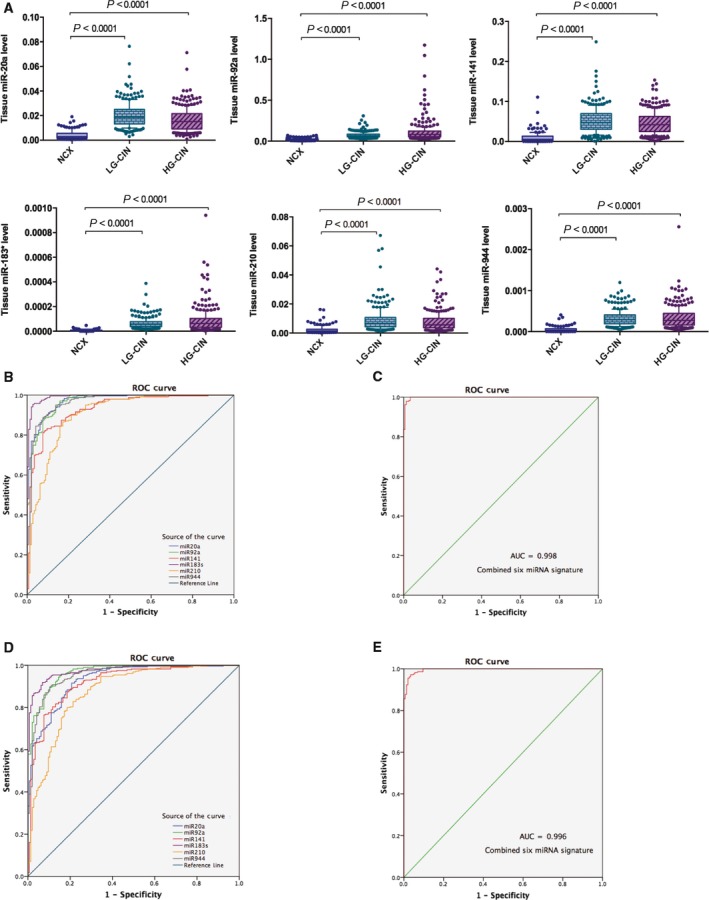
Differential expression of six miRNAs in LG‐CIN, HG‐CIN and normal cervical tissues, and the ROC analyses of single/combined six miRNA detection. (A) Box plot of miRNA expression in tissues (upper: miR‐20a, miR‐92a and miR‐141; lower: miR‐183*, miR‐210 and miR‐944) is normalized to RNU6B. The lines inside the boxes indicate the medians. The boxes mark the interval between the 25th and 75th percentiles. The whiskers and filled circles indicate the interval between and outside the 10th and 90th percentiles. A statistically significant difference is determined by Kruskal–Wallis test with Dunn's multiple comparison post‐hoc test. NCX, normal cervical tissue. (B, C) ROC of six single and combined miRNA detections in LG‐CIN. (D, E) ROC of six single and combined miRNA detections in HG‐CIN.

To determine whether these cervical cancer‐associated miRNA markers have potential clinical significance in the diagnosis and prediction of disease progression in cervical intraepithelial neoplasia, the expression of six miRNA markers was evaluated in CIN samples. The expression patterns of six miRNAs observed in CIN were similar to those in cancer samples. Six miRNAs were significantly up‐regulated in both LG‐CIN and HG‐CIN samples compared to normal controls (*P* < 0.0001 for six miRNAs) (Fig. 4A). The expression of miR‐92a and miR‐183* was increased from LG‐CIN to HG‐CIN, whereas the expression of miR‐20a, miR‐141, miR‐210 and miR‐944 was lower in HG‐CIN compared to LG‐CIN. Aberrant expression of six miRNAs in CIN, especially in low‐grade CIN samples, suggested that these miRNAs may play an important role during the initial or early stages of cervical cancer development.

The diagnostic performance analyses revealed that all six miRNA markers were very good markers in the discrimination of LG‐CIN or HG‐CIN patients from normal individuals (Fig. 4B,D). The AUC values ranged from 0.91 to 0.99 in LG‐CIN and from 0.88 to 0.98 in HG‐CIN. The sensitivity of six miRNAs in the detection of LG‐CIN or HG‐CIN ranged from 83% to 95% and 80% to 92%, respectively, and the specificity ranged from 83% to 97% and 81% to 93% (Table 3). In general, the detection performance of six miRNAs in both LG‐ and HG‐CINs samples was superior to that in cancer samples, except for miR‐141, which showed slightly reduced performance parameters in CIN compared to cancer samples. miR‐183* in CIN samples demonstrated the best performance, showing the highest values of AUC (LG‐CIN 0.99, HG‐CIN 0.98), sensitivity (LG‐CIN 95%, HG‐CIN 92%) and specificity (LG‐CIN 97%, HG‐CIN 92%) among the six miRNAs. In addition, the combined use of miRNA markers further enhanced the sensitivity and specificity of miRNA detection compared to a single miRNA marker (Fig. 4C,E). The multi‐markers achieved an AUC of 0.998 and 0.996, a sensitivity of 97.9% and 97.2%, a specificity of 98.6% and 96.6%, a PPV of 99.2% and 98.2% and a NPV of 96.6% and 94.6% in LG‐ and HG‐CIN samples, respectively. Such high predictive values of the miRNA signature indicated that they could service as a potential multi‐biomarker for the early diagnosis of cervical pre‐malignant lesions and cancer. CIN is a non‐invasive pre‐malignant lesion from which cervical cancer evolves and progresses slowly to the invasive stage. If CIN, particularly low‐grade CIN, can be detected earlier and treated effectively, the incidence of cervical cancer would be significantly reduced.

**Table 3:** Comparison of performance efficiency of miRNA detections as diagnostic markers in LG‐CIN and HG‐CIN

Marker	AUC (95% CI)	Sensitivity (95% CI)	Specificity (95% CI)	PPV % (95% CI)	NPV % (95% CI)
LG‐CIN					
miR‐20a	0.971 (0.957–0.985)	89.1 (84.5–92.8)	91.7 (86.0–95.7)		
miR‐92a	0.967 (0.951–0.984)	88.3 (83.5–92.1)	91.7 (86.0–95.7)		
miR‐141	0.932 (0.905–0.958)	83.3 (77.9–87.8)	90.3 (84.3–94.6)		
miR‐183*	0.992 (0.986–0.999)	95.4 (92.0–97.7)	97.2 (93.1–99.2)		
miR‐210	0.906 (0.873–0.938)	86.6 (81.6–90.7)	82.8 (75.6–88.5)		
miR‐944	0.966 (0.947–0.984)	91.2 (86.9–94.5)	89.7 (83.5–94.1)		
Combined six miRNA signature	0.998 (0.996–1.001)	97.9 (95.2–99.3)	98.6 (95.1–99.8)	99.2 (97.8–1.00)	96.6 (93.3–99.9)
HG‐CIN					
miR‐20a	0.932 (0.908–0.956)	87.7 (83.3–91.3)	82.1 (74.8–87.9)		
miR‐92a	0.967 (0.953–0.982)	89.8 (85.7–93.1)	89.7 (83.5–94.1)		
miR‐141	0.920 (0.892–0.948)	81.8 (76.8–86.1)	86.9 (80.3–91.9)		
miR‐183*	0.976 (0.963–0.988)	91.9 (88.1–94.8)	93.1 (87.7–96.6)		
miR‐210	0.875 (0.839–0.912)	80.0 (74.9–84.5)	81.4 (74.1–87.4)		
miR‐944	0.956 (0.937–0.976)	90.5 (86.5–93.7)	89.0 (82.7–93.6)		
Combined six miRNA signature	0.996 (0.993–0.999)	97.2 (94.5–98.8)	96.6 (92.1–98.9)	98.2 (96.5–99.9)	94.6 (90.4–98.8)

### 3.4 Clinical correlations of six miRNA signature

The potential correlations between six miRNA expression and clinicopathological parameters of CIN and cancer patients were analyzed. The expression levels of miR‐141, miR‐183*, miR‐210 and miR‐944 were significantly higher in younger normal controls (age below mean age) and younger patients with HG‐CIN than in their older age cohorts (normal controls: *P* = 0.002, *P* = 0.007, *P* = 0.002 and *P* < 0.0001; HG‐CIN patients: *P* < 0.0001, *P* = 0.002, *P* = 0.005 and *P* < 0.0001). Similar expression patterns, except for miR‐183*, were also observed in patients with LG‐CIN, although they did not reach statistical significance. However, the expression of miR‐92a was significantly reduced in younger normal controls and younger patients with LG‐CIN (*P* = 0.008 for both). Among the six miRNAs validated, only miR‐944 expression showed a correlation with histological type and tumor grade in cervical cancers. Cancer patients with squamous cell carcinoma or high‐grade (G3) tumor had significantly higher miR‐944 expression (*P* = 0.007 and *P* < 0.0001, respectively) than patients with other cancer cell types or tumors graded at 1–2. Such a correlation was not found for any other miRNA expression. Survival analysis showed no correlation between six miRNA expression and overall or progressive free survival for patients. Hence, the prognostic value of six miRNAs signature in cervical cancer patients is uncertain. This may be a result of the limitation of a relative small size of cancer samples included in the present study, with all of them comprising early stage cancer and 80% of them comprising censored cases during the survival analysis, which may have an impact on the survival correlation study.

### 3.5 miRNA target prediction and function analysis

**Figure 5:**
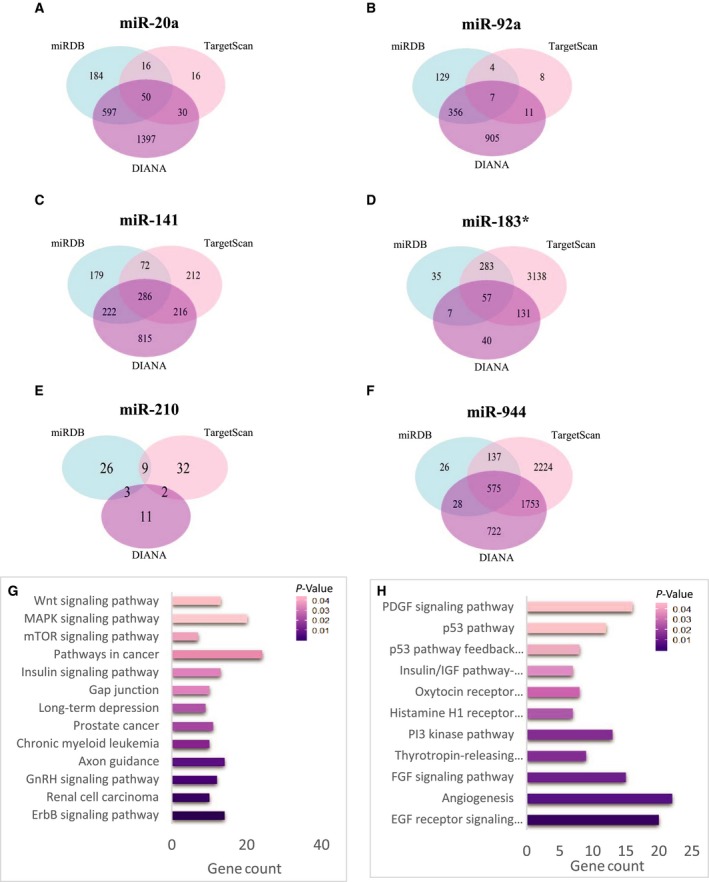
The target gene prediction and function analyses. The Venn diagrams represent the overlapping target genes predicted by miRDB, TargetScan and DIANA_microT‐CDS online analysis tools. (A) miR‐20a; (B) miR‐92a; (C) miR‐141; (D) miR‐183*; (E) miR‐210; (F) miR‐944; (G) the significant enriched KEGG pathways of target genes; and (H) the significant enriched Panther pathways of target genes.

To further understand the functions of six oncogenic miRNAs, the online target gene prediction was performed using three prediction algorithms: miRDB, TargetScan and DIANA_microT‐CDS. The number of overlapping target genes of each miRNA was quite different, from seven to 575 (Fig. 5A–F). Subsequently, pathway enrichment analysis was performed on all overlapping potential target genes of six miRNAs to clarify their potential biological functions. The KEGG (Kyoto Encyclopedia of Genes and Genomes) pathway was significantly enriched in ErbB, GnRH, mTOR, MAPK, Wnt and cancer pathways (Fig. 5G), whereas the Panther (Protein Analysis Through Evolutionary Relationships) pathway was significantly enriched in EGF receptor, angiogenesis, FGF, PI3 kinase, insulin/IGF, MAPK, p53 and PDGF pathways (Fig. 5H). Most of these pathways were critically involved in cancer development and progression, such as cell proliferation, cell cycle, angiogenesis, apoptosis, cell migration and invasion. The results suggested that these six miRNAs were highly associated with cancer. By targeting those oncogenes or tumor suppressor genes, they could regulate the important pathways involved in the initiation and progression of cervical cancer.

**Figure 6:**
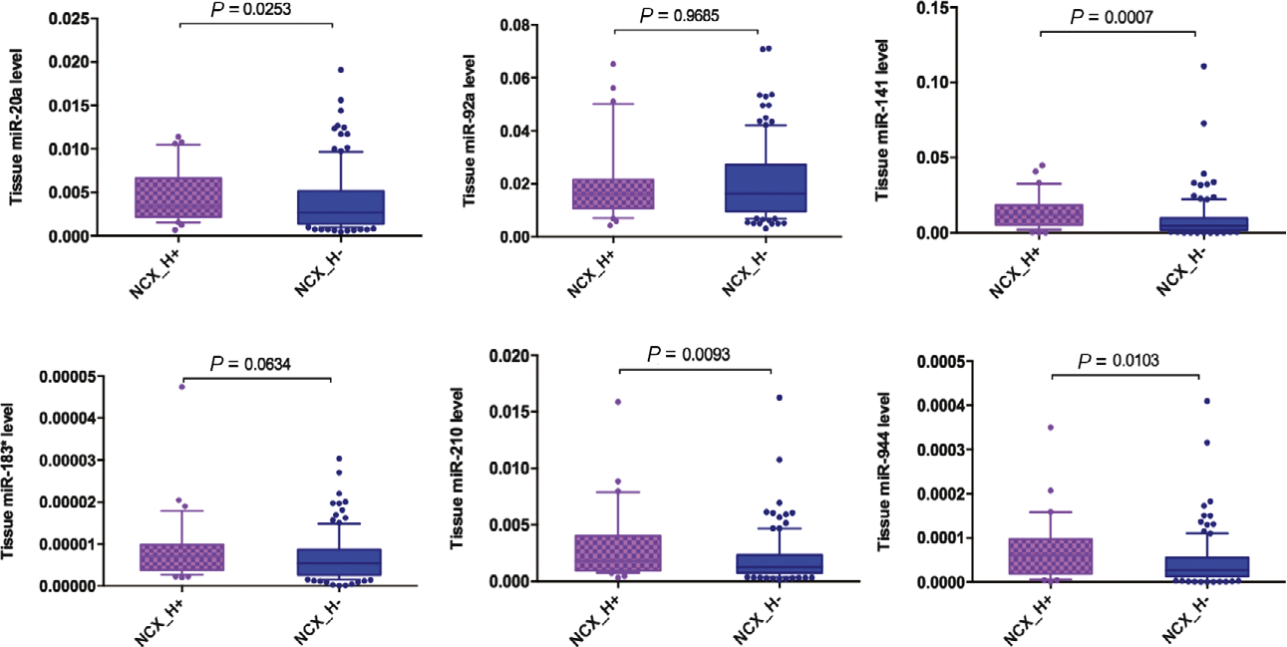
Differential expression of six miRNAs in normal cervical tissues with/without hrHPV infection. Box plot of miRNA expression in normal cervical tissues (upper: miR‐20a, miR‐92a and miR‐141; lower: miR‐183*, miR‐210 and miR‐944) are normalized to RNU6B. The lines inside the boxes indicate the medians. The boxes mark the interval between the 25th and 75th percentiles. The whiskers and filled circles indicate the interval between and outside the 10th and 90th percentiles. A statistically significant difference is determined by the Mann–Whitney test. NCX_H+, normal cervical tissue with hrHPV infection; NCX_H−, normal cervical tissue without hrHPV infection.

To determine whether the expression of six miRNA was associated with hrHPV infection in cervical samples, hrHPV infection was detected in most of cervical cancers (95%), in 82% and 64% of HG‐ and LG‐CINs, and in 24% of normal controls. In normal controls, except miR‐92a, the expression of other five miRNAs was higher in normal cervical tissues with hrHPV infection than in those without infection, and a significant difference was observed in miR‐20a (*P* = 0.025), miR‐141 (*P* < 0.001), miR‐210 (*P* = 0.009) and miR‐944 (*P* = 0.01) (Fig. 6). miRNA expression was not compared between cancer or CIN patients with and without hrHPV infection because the number of patients without HPV infection was disproportionately small for comparison. There are 14 high‐risk HPV types causing cervical cancer, with HPV16 being the most common. Among our hrHPV positive samples, HPV16 was detected in 76% of cervical cancers, 41% of HG‐CINs, 15% of LG‐CINs and 23% of normal controls. The expression of miR‐92a, miR‐183* and miR‐944 was found to be significantly increased in HG‐CIN patients with HPV16 infection compared to patients infected with other high‐risk types of HPV (*P* = 0.002, *P* = 0.045 and *P* = 0.028). Cervical cancer patients with HPV16 infection also showed elevated miR‐944 expression (*P* = 0.055). These results suggested that the aberrant expression of these miRNAs might be the consequence of hrHPV‐induced cervical epithelial cell transformation during the process of HPV infection. hrHPV encoded E6 and E7 oncoproteins are the major oncogenic drivers of cellular transformation. During viral transformation, they also interact with many other multifunctional pathways, such as the miRNA pathway (McLaughlin‐Drubin and Munger, 2009). Therefore, cellular miRNA expression is profoundly influenced by viral infection. A recent deep sequencing study demonstrated that endogenous E6/E7 expression significantly affects the abundance of multiple intracellular miRNAs, such as miR‐17~92 cluster, in HPV‐positive cervical cancer cells, leading to the dysregulation of cell proliferation, senescence and apoptosis (Honegger *et al*., 2015). miR‐20a and miR‐92a are the two important members of miR‐17~92 cluster and were found to be significantly up‐regulated in CINs and cancers in the present study, which was in line with other studies showing the overexpression of these two miRNAs in HPV‐infected cancer and cell lines (Hu *et al*., 2016; Rao *et al*., 2012; Wang *et al*., 2014; Yu *et al*., 2013). In addition, the expression of miR‐141 and miR‐210 was also reported to be regulated by HPV (Wang *et al*., 2014). miR‐141 was found to be up‐regulated in cervical cancer compared to their normal counterparts in other studies (Gao *et al*., 2016; Rao *et al*., 2012). The up‐regulation of miR‐141 in cervical cancer was observed in association with the dysregulation of DROSHA, a critical enzyme in the miRNA biogenesis pathway, for which the expression is suggested to be modulated by HPV16 E6/E7 oncoproteins (Harden and Munger, 2017; Muralidhar *et al*., 2011). miR‐210, is a well‐known hypoxia‐inducible miRNA, termed ‘master hypoxamiRs’. Unlike most hypoxamiRs, miR‐210 shows a high and consistent up‐regulation in many human cancer types, including cervical cancer (Rao *et al*., 2012). Its expression was found to be significantly higher in HPV‐positive than in HPV‐negative cervical cancers and cell lines (Wang *et al*., 2014). It has been reported that miR‐210 promoted cell proliferation and cell cycle progression by directly targeting MNT, a known MYC antagonist, leading to the activation of c‐MYC in HPV transformed cells (Zhang *et al*., 2009). It is well documented that MYC promotes cell proliferation in cervical pre‐malignant lesions cancer. The diagnostic and prognostic values of miR‐210 as biomarker have been demonstrated in various human cancers (Li *et al*., 2014; Qin *et al*., 2014).

The expression of miR‐183* and miR‐944 in various human cancers was quite controversial. These miRNAs function as either an oncogene or tumor suppressor depending on tumor context and cell types. Most of studies of miR‐183 focus on the expression and function of miR‐183‐5p, and only one recent study reported the up‐regulated expression of miR‐183* in lung adenocarcinoma (Xu *et al*., 2014). miR‐183‐5p and miR‐183* are the forward and reverse miRNA stands of a miRNA duplex, respectively. It has been shown that both stands of miRNA could be expressed in specific tissues/cells and that they might have functional relevance (Witten *et al*., 2010). The present study showed that miR‐183* expression was significantly up‐regulated in CIN and cancer tissue samples compared to normal cervical tissue samples, suggesting the oncogenic nature of miR‐183*. miR‐944 was first identified in human cervical cells using a small RNA cloning approach (Lui *et al*., 2007). It is located in the intron of TP63 (a member of the p53 family) and maps to chromosome 3q27‐28, a region frequently amplified in cervical cancer (Narayan *et al*., 2007). It was found to be co‐overexpressed with p63 gene in cervical cancer and its transcription is dependent on the transactivating role of ΔNp63 within the promoter region (Xie *et al*., 2015). miR‐944 was found to be significantly abundant in cervical cancer tissues compared in their normal counterparts (Witten *et al*., 2010; Xie *et al*., 2015), as well as in sera of women with metastatic cervical cancer (Palatnik *et al*., 2017).

## 4 Conclusions

The present study identified a unique six miRNA signature (miR‐20a, miR‐92a, miR‐141, miR‐183*, miR‐210 and miR‐944) with oncogenic potential, and some of these miRNAs are associated with hrHPV infection in cervical pre‐malignant lesions and cancer. Because their expression is associated with different stages of cervical cancer development, this will enable the better understanding of the underlying molecular mechanisms involved in the cervical carcinogenesis in future studies. More importantly, this miRNA signature offers a superior diagnostic value, plus an extremely high sensitivity and specificity, in patients with cervical pre‐malignant lesions and cancer. Because both cytology and HPV detection show limitations as stand‐alone screening tests, miRNA detection may provide a potential screening option in cervical cancer screening. Nevertheless, further investigations conducted in a large clinical‐based population in comparison with cytology and HPV screening methods are needed to assess our findings before any clinical application of this miRNA signature.

## Author contributions

SL, EN and HN contributed to conception and design of study. SL and KC supervised the study. SL and DL performed the experiments. KC, SN, MC, KT, PI and AC recruited clinical samples. TW and LL retrieved clinical data and contributed in statistical analyses. SL interpreted and analyzed data and wrote the manuscript. All authors have approved the final version of the manuscript submitted for publication.

## Supporting information


**Fig. S1.** Expressions of RNU6B, miR‐25‐3p and miR‐93‐5p in tissues from normal control, CIN and cervical cancer patients.
